# Synthesis and preclinical evaluation of DOTAGA-conjugated PSMA ligands for functional imaging and endoradiotherapy of prostate cancer

**DOI:** 10.1186/s13550-014-0063-1

**Published:** 2014-11-25

**Authors:** Martina Weineisen, Jakub Simecek, Margret Schottelius, Markus Schwaiger, Hans-Jürgen Wester

**Affiliations:** Pharmaceutical Radiochemistry, Technical University Munich, Walther-Meißner-Str. 3, 85748 Garching, Germany; Department of Nuclear Medicine, Technical University Munich (Klinikum rechts der Isar), Ismaninger Straße 22, 81675 München, Germany

**Keywords:** Prostate cancer, Theranostic, Endoradiotherapy, PET, PSMA, Prostate-specific membrane antigen, PSMA ligand, [^68^Ga]DOTAGA-ffk(Sub-KuE), [^177^Lu]DOTAGA-ffk(Sub-KuE)

## Abstract

**Background:**

Due to its high expression in prostate cancer, PSMA (prostate-specific membrane antigen) represents an ideal target for both diagnostic imaging and endoradiotherapeutic approaches. Based on a previously published highly specific PSMA ligand ([^68^Ga]DOTA-FFK(Sub-KuE)), we developed a corresponding metabolically stable 1,4,7,10-tetraazacyclododececane,1-(glutaric acid)-4,7,10-triacetic acid (DOTAGA) construct for theranostic treatment of prostate cancer.

**Methods:**

All ligands were synthesized by a combined solid phase and solution phase synthesis strategy. The affinity of the ^nat^gallium and lutetium complexes to PSMA and the internalization efficiency of the radiotracers were determined on PSMA-expressing LNCaP cells. The ^68^Ga- and ^177^Lu-labelled ligands were further investigated for lipophilicity, binding specificity, metabolic stability, as well as biodistribution and μPET in LNCaP-tumour-bearing mice.

**Results:**

Radiochemical yields for ^68^Ga (3 nmol, 5.0 M NaCl/2.7 M HEPES (approximately 5/1), pH 3.5 to 4.5, 5 min, 95°C) and ^177^Lu labelling (0.7 nmol, 0.1 M NH_4_OAc, pH 5.5, 30 min, 95°C) were almost quantitative, resulting in specific activities of 250 to 300 GBq/μmol for the ^68^Ga analogues and 38 GBq/μmol for ^177^Lu complexes. Due to metabolic instability of l-amino acid spacers, d-amino acids were implemented resulting in a metabolically stable DOTAGA ligand. Compared to the DOTA ligand, the DOTAGA derivatives showed higher hydrophilicity (log*P* = −3.6 ± 0.1 and −3.9 ± 0.1 for ^68^Ga and ^177^Lu, respectively) and improved affinity to PSMA resulting in an about twofold increased specific internalization of the ^68^Ga- and ^177^Lu-labelled DOTAGA analogue. Especially, [^68^Ga]DOTAGA-ffk(Sub-KuE) exhibits favourable pharmacokinetics, low unspecific uptake and high tumour accumulation in LNCaP-tumour-bearing mice.

**Conclusions:**

The pair of diagnostic/therapeutic PSMA-ligands [^68^Ga/^177^Lu]DOTAGA-ffk(Sub-KuE) possess remarkable potential for the management of prostate cancer.

## Background

Prostate-specific membrane antigen (PSMA, EC 3.4.17.21, synonym: glutamate carboxypeptidase II) is an extracellular hydrolase whose catalytic centre comprises two zinc(II) ions with a bridging hydroxido ligand [1]. It is highly up-regulated in metastatic and hormone-refractory prostate carcinomas, but its endogenous expression has also been reported in kidneys, salivary glands, small intestine, brain and, to a low extent, also in healthy prostate tissue [2,3]. In the intestine, PSMA facilitates absorption of folate by conversion of pteroylpoly-γ-glutamate to the pteroylglutamate (folate) [4]. In the brain, it hydrolyses *N*-acetyl-l-aspartyl-l-glutamate (NAAG) to *N*-acetyl-l-aspartate and glutamate [5]. The enzymatic function of PSMA in normal and diseased prostate has not been clarified yet [6]. However, due to its overexpression on prostate cancer cells, PSMA represents an excellent target for molecular imaging and targeted radiotherapy of prostate cancer.

**Figure 1:**
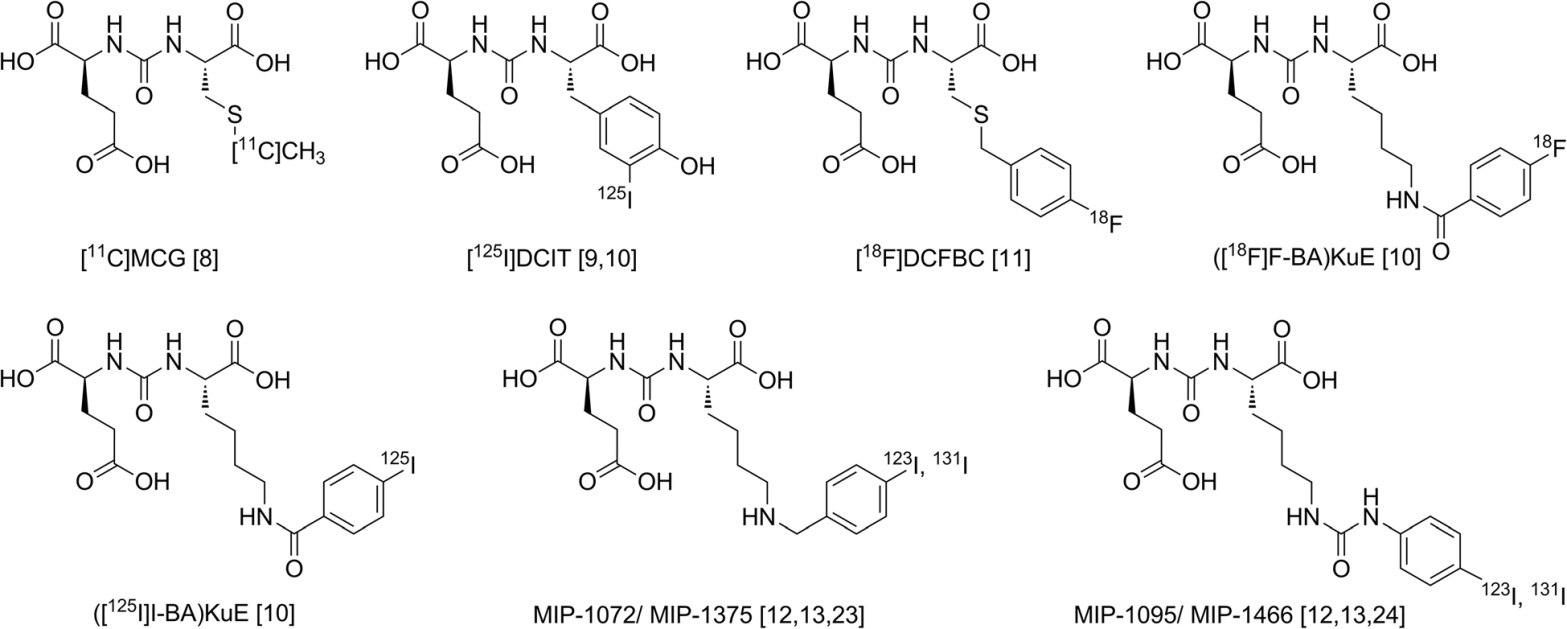
**Structures of selected PSMA ligands.** Depicted are the first ^11^C-labelled and radiohalogenated PSMA ligands based on the XuE-scaffold (*X* = *C*, *Y*, *K* or *E*).

Since the discovery of urea-based PSMA inhibitors in 2001 [7], a variety of PSMA-targeted radioligands for imaging of prostate cancer were developed. The first PSMA inhibitors radiolabelled with ^11^C, ^18^ F and ^123/125^I used the X-urea-Glu (XuE)-scaffold (Figure 1, [8-13]). All of them show high affinity to PSMA and specific tumour accumulation, demonstrating the suitability of this class of compounds as imaging probes. Subsequently, ^99m^Tc-labelled analogues [14,15] were developed to provide a generator produced PSMA imaging agent for SPECT.

Recently, it has been demonstrated that the affinity of KuE-derived inhibitors is enhanced by extending the KuE-binding unit by an aromatic moiety, presumably due to an additional inhibitor-enzyme interaction via π/π-stacking of the additional aromatic residues with a remote arene-binding pocket [16]. There is apparently an optimal distance between the KuE-inhibitor component and the additional arene moiety, leading to an enhancement of PSMA affinity (*K*_*D*_ = 13.8 nM) by a factor of up to 5 [15].

**Figure 2:**
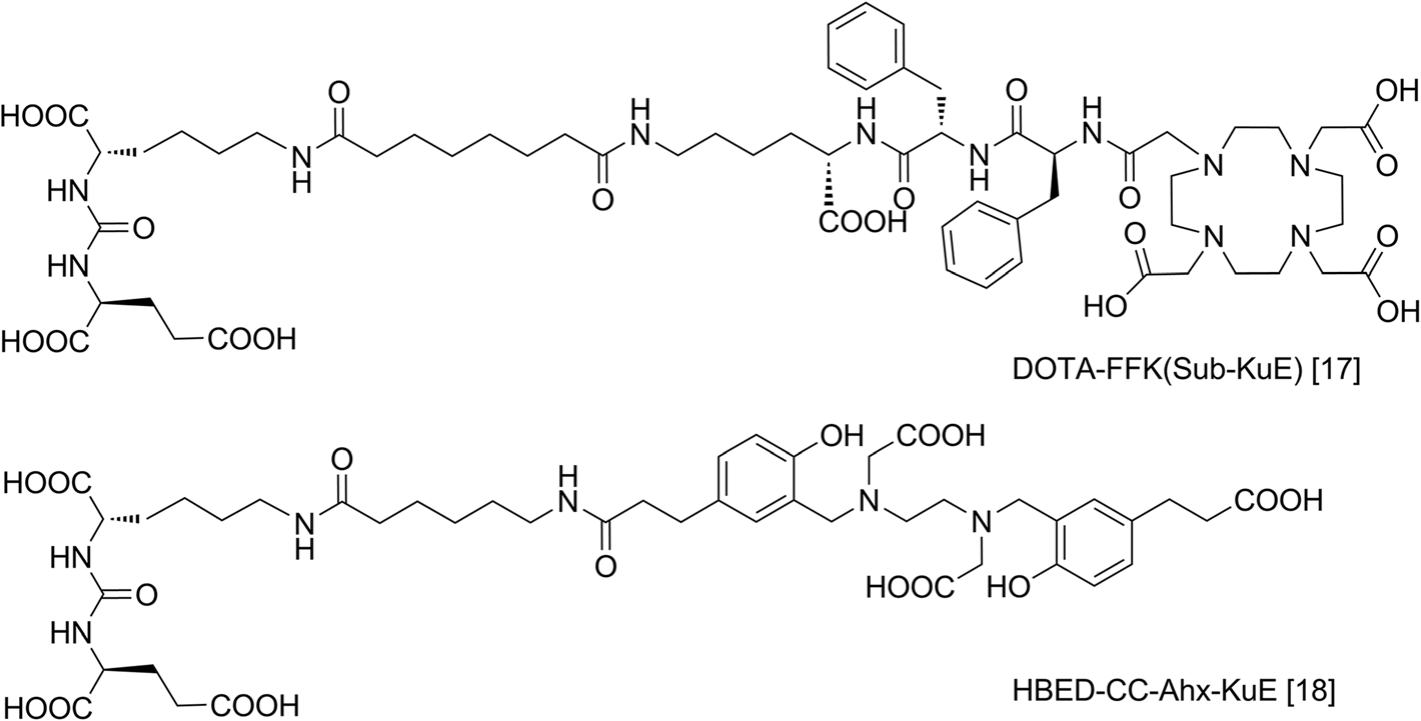
**Previously reported PSMA ligands for** ^**68**^ **Ga labelling** **[** 17,18 **].** Both compounds were included as references in this study.

These findings were recently integrated into the design of ^68^Ga-labelled PSMA ligands (Figure 2), such as [^68^Ga]DOTA-FFK(Sub-KuE) [17] and [^68^Ga]HBED-CC-Ahx-KuE [18]. Compared to [^68^Ga]DOTA-FFK(Sub-KuE), [^68^Ga]HBED-CC-Ahx-KuE showed higher tumour accumulation and improved imaging contrast [18]. Unfortunately, the HBED-CC chelator (*N*,*N*′-bis[2-hydroxy-5-(carboxyethyl)benzyl] ethylenediamine-*N*,*N*′-diacetic acid) is not suitable for radiolabelling with therapeutic radiometals, whereas 1,4,7,10-tetraazacyclododecane-1,4,7,10-tetraacetic acid (DOTA) and its analogues form stable complexes with a broad range of radiometals, for therapy most importantly ^90^Y and ^177^Lu. Given the high expression of PSMA on the majority of prostate cancers, the availability of high-affinity PSMA-targeted probes, labelled with therapeutic radioisotopes, offers promising perspectives for PSMA-targeted endoradiotherapy.

Monoclonal anti-PSMA-antibodies labelled with therapeutic radionuclides such as ^131^I, ^90^Y and ^177^Lu, which are currently being evaluated in clinical trials (clinicaltrials.gov Identifier: NCT00006380, NCT00081172) already demonstrate promising tumour targeting, acceptable toxicity and therapeutic efficiency in humans [19-22].

Amongst the currently available small-molecule PSMA inhibitors, only [^131^I]MIP-1375 [23] and [^131^I]MIP-1466 [24] (Figure 1) have been evaluated in therapy studies so far. In LNCaP-tumour-bearing nude mice, [^131^I]MIP-1375 inhibited tumour growth in a time- and dose-dependent manner. First, human applications of [^131^I]MIP-1095/1466 revealed promising therapy response with moderate side effects [25]. The structurally corresponding diagnostics, ^123^I-labelled analogues [^123^I]MIP-1072 and [^123^I]MIP-1095, were shown to possess suitable characteristics for SPECT imaging. However, these compounds are comparably lipophilic (clog*P* = 0.20 and 1.97, respectively [25]), and their compact structure complicates further optimization of PSMA affinity and pharmacokinetics. Furthermore, PET imaging is only possible using ^124^I, a suboptimal PET-radionuclide with respect to resolution and dosimetry.

In contrast, radiometalated analogues based on the FFK(Sub-KuE)-scaffold represent a much more flexible and finely adjustable backbone for the development of KuE-based PSMA inhibitors, that allow labelling with both diagnostic and therapeutic radionuclides, e.g. ^68^Ga for PET imaging and ^177^Lu/^90^Y for therapy after conjugation of DOTA. However, in order to further facilitate the labelling procedure for ^177^Lu and ^90^Y, improve ligand pharmacokinetics, and potentially obtain radiometalated derivatives with higher affinity, we substituted DOTA in DOTA-FFK(Sub-KuE) by 1,4,7,10-tetraazacyclodocecane,1-(glutaric acid)-4,7,10-triacetic acid (DOTAGA) [26,27]. Improved affinities, higher tumour uptake and faster kidney clearance have already been observed for the ^68^Ga-complex (one free carboxylate) compared to the ^90^Y-complex (all carboxylates coordinated) of DOTA-coupled somatostatin analogues before [28].

Consequently, we evaluated and compared the respective ^68/nat^Ga- and ^177/nat^Lu-DOTAGA analogues of FFK(Sub-KuE) and ffk(Sub-KuE) in terms of PSMA affinity, uptake in PSMA positive tumour cells, metabolic stability, in vivo biodistribution and μPET imaging. The previously described DOTA analogues [17] and [^68^Ga]HBED-CC-Ahx-KuE [18] were also included to allow direct comparability of our results with the data in the literature.

## Methods

### General

Fmoc-(9-fluorenylmethoxycarbonyl-) and all other protected amino acid analogues were purchased from Iris Biotech (Marktredwitz, Germany) or Bachem (Bubendorf, Switzerland). Tritylchloride polystyrene (TCP) resin was obtained from PepChem (Tübingen, Germany). The chelators DOTA-tris-*t*Bu-ester and DOTAGA-anhydride were purchased from Chematech (Dijon, France). Solvents and all other organic reagents were purchased from Sigma-Aldrich (Munich, Germany) or CLN (Freising, Germany). Solid phase peptide synthesis was carried out manually using an Intelli-Mixer syringe shaker (Neolab, Heidelberg, Germany). Analytical reversed-phase high performance liquid chromatography (RP-HPLC) was performed on a Nucleosil 100 C18 (5 μm, 125 × 4.0 mm) column (CS GmbH, Langerwehe, Germany) using a Sykam gradient HPLC System (Sykam GmbH, Eresing, Germany). The peptides were eluted applying different gradients of 0.1% (*v*/*v*) trifluoroacetic acid (TFA) in H_2_O (solvent A) and 0.1% TFA (*v*/*v*) in acetonitrile (solvent B) at a constant flow of 1 mL/min (specific gradients are cited in the text). UV detection was performed at 220 nm using a 206 PHD UV-Vis detector (Linear™ Instruments Corporation, Reno, USA). Both retention times *t*_*R*_ as well as the capacity factors *K*' are cited in the text. Preparative RP-HPLC was performed on the same HPLC system using a Multospher 100 RP 18-5 (250 × 20 mm) column (CS GmbH, Langerwehe, Germany) at a constant flow of 9 mL/min. Radio-HPLC of the radioiodinated reference ligand was carried out using a Nucleosil 100 C18 (5 μm, 125 × 4.0 mm) column. For radioactivity detection, the outlet of the UV-photometer was connected to a NaI(Tl) well-type scintillation counter from EG&G Ortec (Munich, Germany). Analysis of ^68^Ga-labelled compounds was done as described previously [29]. ESI-mass spectra were acquired on a Varian 500-MS IT mass spectrometer (Agilent Technologies, Santa Clara, USA).

### Synthesis of carboxyl-protected Lys-urea-Glu-core (KuE)

(S)**-**di**-**tert**-**butyl 2**-**(1H**-**imidazole**-**1**-**carboxamido)pentanedioate (**1**) was synthesized as described previously [12]. HPLC (10% to 90% B in 15 min): *t*_*R*_ = 12.2 min; *K*′ = 5.78. Calculated monoisotopic mass for **1** (C_17_H_27_N_3_O_5_): 353.4; found: *m*/*z* = 376.0 [M + Na]^+^.

Cbz**-**(OtBu)KuE(OtBu)_2_ (**2**): A solution of 3.40 g (9.64 mmol, 1.0 eq) **1** in 45 mL 1,2-dichloroethane (DCE) was cooled to 0°C, and 2.69 mL (19.28 mmol, 2.0 eq) of triethylamine (TEA), and 3.59 g (9.64 mmol, 1.0 eq) of Cbz-Lys-OtBu · HCl was added under vigorous stirring. The reaction mixture was heated to 40°C overnight. The solvent was removed *in vacuo*, and the crude product was purified via silica gel flash-chromatography using an eluent mixture of ethyl acetate/hexane/TEA (500/500/0.8 (v/v/v)). Upon solvent evaporation, 4.80 g of **2** were obtained as a colourless, sticky oil (yield: 80% based on l-di-tert-butyl glutamate · HCl). HPLC (40% to 100% B in 15 min): *t*_*R*_ = 14.3 min; *K*′ = 8.53. Calculated monoisotopic mass for **2** (C_32_H_51_N_3_O_9_): 621.8; found: *m*/*z* = 622.2 [M + H]^+^, 644.3 [M + Na]^+^.

(OtBu)KuE(OtBu)_2_ (**3**): For Cbz deprotection, 6.037 g (9.71 mmol, 1.0 eq) of **2** was dissolved in 150 mL of ethanol (EtOH), and 0.6 g (1.0 mmol, 0.1 eq) of Palladium on activated charcoal (10%) was added. After purging the flask with H_2_, the solution was stirred overnight under light H_2_-pressure (balloon). The crude product was filtered through Celite, the solvent was evaporated *in vacuo*, and the desired product was obtained as a waxy solid (4.33 g, 91.5% yield). HPLC (10% to 90% B in 15 min): *t*_*R*_ = 12.6 min; *K*′ = 6.41. Calculated monoisotopic mass for **3** (C_24_H_45_N_3_O_7_): 487.6; found: *m*/*z* = 488.3 [M + H]^+^, 510.3 [M + Na]^+^.

### Synthesis of protected Sub-KuE conjugate

NHS**-**Sub**-**(OtBu)KuE(OtBu)_2_ (**4**): **3** (40 mg, 0.08 mmol, 1 eq) was dissolved in 500 μL *N*,*N*-dimethylformamide (DMF), and 57 μL (0.41 mmol, 5 eq) of TEA was added. This solution was added dropwise (within 30 min) to a solution of 33.2 mg (0.09 mmol, 1.1 eq) of disuccinimidyl suberate (DSS). After stirring for an additional 2 h at room temperature (RT), the reaction mixture was concentrated *in vacuo*, diluted with ethyl acetate and extracted with water (twice). The organic phase was dried over Na_2_SO_4_, filtered and evaporated to dryness. Due to sufficient purity of the crude **4**, it was used for the following reaction step without further purification. HPLC (10% to 90% B in 15 min): *t*_*R*_ = 16.9 min; *K*′ = 8.39. Calculated monoisotopic mass for **4** (C_36_H_60_N_4_O_12_): 740.4; found: *m*/*z* = 741.2 [M + H]^+^, 763.4 [M + Na]^+^.

### Synthesis of peptidic spacers

Fmoc**-**l**-**Phe**-**l**-**Phe**-**l**-**Lys(Boc) (Fmoc**-**FFK, **5**) and Fmoc**-**d**-**Phe**-**d**-**Phe**-**d**-**Lys(Boc) (Fmoc**-**ffk, **6**): Fmoc-Lys(Boc)-OH was coupled to TCP resin according to a previously published method [30]. Briefly, Fmoc-Lys(Boc)-OH (1.5 eq) was dissolved in dry dichloromethane (DCM), and *N*,*N*-diisopropylethylamine (DIPEA) (1.25 eq) was added. Dry TCP resin was suspended and stirred at RT for 5 min. Another 2.5 eq of DIPEA was added, and stirring was continued for 90 min. Then, 1 mL methanol (MeOH) per gram resin was added to cap unreacted Tritylchloride groups. After 15 min, the resin was filtered off, washed twice with DCM, DMF and MeOH, respectively, and dried *in vacuo*. Final load of resin-bound Fmoc-Lys(Boc)-OH was calculated from the weight difference.

Assembly of the peptide sequence H_2_N-Phe-Phe- on resin-bound Lys(Boc) was performed according to a standard Fmoc-protocol using 1.5 eq of 1-hydroxybenzotriazole (HOBt) and *O*-(1H-benzotriazol-1-yl)-*N*,*N*,*N*’,*N*’-tetramethyluronium-tetrafluoroborate (TBTU) as coupling reagents and 4.5 eq DIPEA. After coupling of the last amino acid, the resin was washed, dried and stored in a desiccator until further functionalization.

### Coupling of chelating moiety

Fmoc-Phe-Phe-Lys(Boc)-TCP resin was allowed to preswell in *N*-methyl-pyrrolidon (NMP) for 30 min. After cleavage of the N-terminal Fmoc-protecting group using 20% piperidine in DMF (*v*/*v*), the resin was washed eight times with NMP. The coupling of the respective chelators is described below. Cleavage from the resin (2 × 30 min) and concomitant *t*Bu-deprotection was performed using a mixture (*v*/*v*/*v*) of 95% TFA, 2.5% triisobutylsilane (TIBS) and 2.5% water. The combined product solutions were then concentrated, the crude peptide was precipitated using diethyl ether and was dried *in vacuo*. Due to sufficient purity of the crude products, they were used for the following reaction step without further purification.

DOTA**-**Phe**-**Phe**-**Lys (DOTA**-**FFK, **7**) [17]: For 38 μmol of resin-bound peptide, 33 mg of DOTA-tris-*t*Bu-ester (57 μmol, 1.5 eq), 108 mg of O-(7-azabenzotriazol-1-yl)-*N*,*N*,*N*′,*N*′-tetramethyluronium hexafluorophosphate (HATU; 0.28 μmol, 5 eq) and 87 μL of DIPEA (570 μmol, 15 eq) in NMP were added to the resin. After 72 h of shaking, the resin was washed with NMP and DCM. HPLC (10% to 90% B in 15 min): *t*_*R*_ = 8.2 min; *K*′ = 4.13. Calculated monoisotopic mass for **7** (C_40_H_58_N_8_O_11_): 826.4; found: *m*/*z* = 827.3 [M + H]^+^, 849.3 [M + Na]^+^, 414.2 [M +2H]^2+^.

DOTAGA**-**Phe**-**Phe**-**Lys (DOTAGA**-**FFK, **8** and DOTAGA**-**ffk, **9**): For 0.27 mmol peptide-bound resin, 190 mg DOTAGA-anhydride (0.42 mmol, 1.5 eq) and 470 μL DIPEA (2.7 mmol, 10 eq) in NMP were added to the resin. After 18 h of shaking, the resin was washed with NMP and DCM. HPLC (10% to 90% B in 15 min): *t*_*R*_ = 10.6 min; *K*′ = 5.63. Calculated monoisotopic mass for **8** and **9** (C_43_H_62_N_8_O_13_): 898.4; found: *m*/*z* = 899.4 [M + H]^+^, 921.4 [M + Na]^+^, 450.2 [M +2H]^2+^.

### Condensation of the chelator-conjugated peptides and the PSMA binding motif

DOTA**-**FFK(Sub**-**KuE) (**10**) [17]: To a solution of **7** (15 mg, 18 μmol, 1 eq) and TEA (13 μL, 90 μmol, 5 eq) dissolved in 600 μL of DMF was slowly added 13 mg of **4** (18 μmol, 1 eq) dissolved in 400 μL of DMF. After stirring for 2 h at RT, the reaction mixture was evaporated to dryness. Subsequent removal of *t*Bu-protecting groups was carried out by dissolving the crude product in TFA and stirring for 40 min. After precipitation in diethyl ether, the crude product was dissolved in water and purified using preparative RP-HPLC (25% to 40% B in 20 min). HPLC (10% to 90% B in 15 min): *t*_*R*_ = 10.3 min; *K*′ = 5.44. Calculated monoisotopic mass for **10** (C_60_H_89_N_11_O_20_): 1,283.6; found: *m*/*z* = 1,284.5 [M + H]^+^, 1,306.7 [M + Na]^+^, 642.8 [M +2H]^2+^.

DOTAGA**-**FFK**(**Sub**-**KuE) (**11**) and DOTAGA**-**ffk(Sub**-**KuE) (**12**): Either **8** or **9** (21 mg, 30 μmol, 1 eq) was added to TEA (21 μL, 150 μmol, 5 eq) and 27 mg of **4** (30 μmol, 1 eq) as described for **10**. HPLC (10% to 90% B in 15 min): *t*_*R*_ = 9.7 min; *K*′ = 4.11. Calculated monoisotopic mass for **11** and **12** (C_63_H_93_N_11_O_22_): 1,355.7; found: *m*/*z* = 1,356.2 [M + H]^+^, 1,378.2 [M + Na]^+^, 679.2 [M +2H]^2+^.

### Synthesis of the radioiodination precursor **(13)**

The synthesis was performed according to previously published methods [12,31,32].

Succinimidyl**-**4**-**iodobenzoate (I**-**BA**-**NHS, **14**): Under a nitrogen atmosphere, 500 mg (2.0 mmol, 1.0 eq) 4-iodobenzoic acid was dissolved in 10 mL DCM, and after addition of 278 mg (2.4 mmol, 1.2 eq) *N*-hydroxysuccinimide and 374 mg (1.81 mmol, 0.9 eq) dicyclohexyl carbodiimide, the suspension was stirred overnight. The precipitate was filtered off, and the filtrate was evaporated to dryness. The resulting solid was washed with a 1:1-mixture of DCM and hexane to yield the desired product (583.5 mg, 93%) as a white solid. Due to the limited detectability of the product in ESI-mass spectrometry (MS), a representative conjugate with H-Phe-OtBu (1 eq) was prepared in DMF in the presence of DIPEA (3 eq) and characterized via MS. HPLC (40% to 100% B in 15 min): *t*_*R*_ = 10.6 min; *K*' = 5.63. Calculated monoisotopic mass for I-BA-Phe(OtBu) (C_20_H_22_INO_3_): 451.1; found: *m*/*z* = 396.1 [M + H-tBu]^+^.

Succinimidyl**-**4**-**tributylstannyl**-**benzoate **(**SnBu_3_**-**BA**-**NHS, **15**): To a solution of 100 mg (0.29 mmol, 1.0 eq) **14** in 5 mL anhydrous toluene were added 234 μL (0.464 mmol, 1.6 eq) of hexabutylditin and 10.7 mg (9 μmol, 0.02 eq) of the catalyst *tetrakis*(triphenylphosphine)palladium under a nitrogen atmosphere. The mixture was heated under reflux until the solution turned black (overnight). After cooling, the toluene was removed *in vacuo*, and the resulting oil was purified using silica gel flash chromatography (ethyl acetate/hexane: 3/7 (v/v)) to yield **15** (78 mg, 53%) as a colourless oil. TLC (ethyl acetate/hexane: 3/7): *R*_*f*_ = 0.46

(SnBu_3_**-**BA)(OtBu)KuE(OtBu)_2_ (**13**): To a solution of 19.0 mg (0.039 mmol, 1.0 eq) **3** in 2 mL DCM were added 26.3 μL (0.187 mmol, 4.8 eq) of TEA and 19.8 mg (0.039 mmol, 1.0 eq) of **15**. The mixture was stirred at RT for 4 h and was then diluted with DCM. After washing with water, the organic phase was dried over Na_2_SO_4_, filtered and evaporated to dryness. **13** (30.8 mg, 89.7%) was obtained as a colourless oil. HPLC (10% to 90% B in 15 min): *t*_*R*_ = 23.8 min; *K*' = 13.88. Calculated monoisotopic mass for **13** (C_43_H_75_N_3_O_8_Sn): 880.8; found: *m*/*z* = 902.2/903.3/904.3 [M + Na]^+^.

### Synthesis of unlabelled reference compounds

**(**I**-**BA**)**KuE: To a solution of 15 mg (0.031 mmol, 1.0 eq) **3** in 2 mL DMF was added 11.4 mg (0.046 mmol, 1.5 eq) of 4-iodo-benzoic acid, 6.3 mg (0.046 mmol, 1.5 eq) of 1-Hydroxy-7-azabenzotriazole (HOAt), 7.2 μL (0.046 mmol, 1.5 eq) diisopropyl carbodiimide (DIC) and 23.7 μL (0.138 mmol, 4.5 eq) DIPEA. The yellow solution was stirred for 20 h, diluted with ethyl acetate and extracted with water. The organic layer was dried over Na_2_SO_4_, filtered and evaporated to dryness. For *t*Bu-deprotection, the crude product was dissolved in 200 μL TFA. After 30 min, the solvent was evaporated and the crude product was purified using preparative RP-HPLC (isocratic eluent: 18% B). HPLC (10% to 90% B in 15 min): *t*_*R*_ = 10.5 min; *K*′ = 5.56 calculated monoisotopic mass for (I-BA)KuE (C_19_H_24_IN_3_O_8_): 549.3; found: *m*/*z* = 550.0 [M + H]^+^, 571.9 [M + Na]^+^

^*nat*^*Ga compounds*: For the preparation of the ^nat^Ga complexes, equal volumes of a 2 mM solution of Ga(NO_3_)_3_ in water and a 2 mM solution of the respective PSMA ligand in water were mixed and heated to 40°C for 30 min. After cooling, the ^nat^Ga^III^-chelate formation was confirmed using RP-HPLC and MS. The resulting 1 mM aqueous solutions of the respective ^nat^Ga-complexes were then further diluted and used in the *in vitro* IC_50_ studies without further processing.

[^nat^Ga]DOTA-FFK(Sub-KuE) (^nat^Ga-**10**) HPLC (20% to 60% B in 15 min): *t*_*R*_ = 11.6 min; *K*′ = 6.3. Ccalculated monoisotopic mass (C_60_H_86_N_11_O_20_Ga): 1,349.5; found: *m*/*z* = 1,350.3 [M + H]^+^, 1,372.1 [M + Na]^+^, 675.7 [M +2H]^2+^.

[^nat^Ga]DOTAGA-FFK(Sub-KuE) (^nat^Ga-**11**) HPLC (25% to 45% B in 15 min): *t*_*R*_ = 16.0 min; *K*′ = 9.0. Calculated monoisotopic mass (C_63_H_90_N_11_O_22_Ga): 1,421.7, 1,423.7; found: *m*/*z* = 1,422.1/1,424.1 [M + H]^+^, 710.6/712.6 [M +2H]^2+^.

[^nat^Ga]DOTAGA-ffk(Sub-KuE) (^nat^Ga-**12**) HPLC (25% to 55% B in 15 min): *t*_*R*_ = 12.1 min; *K*′ = 7.6. Calculated monoisotopic mass (C_63_H_90_N_11_O_22_Ga): 1,421.7, 1,423.7; found: *m*/*z* = 1,422.6/1,424.5 [M + H]^+^, 1,444.4/1,446.4 [M + Na]^+^.

[^nat^Ga]HBED-CC-Ahx-KuE HPLC (25% to 43% B in 15 min): *t*_*R*_ = 9.0 min; *K*′ = 5.0. Calculated monoisotopic mass (C_44_H_59_N_6_O_17_Ga): 1,012.3, 1,014.3; found: *m*/*z* = 1,013.0/1,015.0 [M + H]^+^, 1,035.0/1,037.0 [M + Na]^+^.

^*nat*^*Lu****-****compounds*: The corresponding ^nat^Lu^III^ complexes were prepared with a 2.5-molar excess of Lu^3+^, heated at 95°C for 30 min and evaluated similar to the ^nat^Ga^III^ complexes.

[^nat^Lu]DOTA-FFK(Sub-KuE) (^nat^Lu-**10**) HPLC (25% to 45% B in 16 min): *t*_*R*_ = 14.1 min; *K*′ = 9.1. Calculated monoisotopic mass (C_60_H_86_N_11_O_20_Lu): 1,455.6; found: *m*/*z* = 1,456.4 [M + H]^+^, 1,478.5 [M + Na]^+^.

[^nat^Lu]DOTAGA-FFK(Sub-KuE) (^nat^Lu-**11**) HPLC (25% to 45% B in 16 min): *t*_*R*_ = 14.4 min; *K*′ = 9.3. Calculated monoisotopic mass (C_63_H_90_N_11_O_22_Lu): 1,527.6; found: *m*/*z* = 1,528.4 [M + H]^+^, 1,550.3 [M + Na]^+^, 764.2 [M +2H]^2+^.

[^nat^Lu]DOTAGA-ffk(Sub-KuE) (^nat^Lu-**12**) HPLC (25% to 55% B in 15 min): *t*_*R*_ = 10.4 min; *K*′ = 6.4. Calculated monoisotopic mass (C_63_H_90_N_11_O_22_Lu): 1,527.6; found: *m*/*z* = 1,528.1 [M + H]^+^, 764.5 [M +2H]^2+^.

### Radiolabelling

([^*125*^*I*]*I****-****BA*)*KuE*: The radioiodination was performed according to previously published methods [12,31,32]. Peracetic acid was prepared by mixing 130 μL of H_2_O_2_ (30%) and 50 μL of acetic acid. After a 2 h-incubation period, 20 μL peracetic acid solution and 5 μL (21.0 MBq) [^125^I]NaI (74 TBq/mmol, 3.1 GBq/mL 40 mM NaOH, Hartmann Analytic, Braunschweig, Germany) was added to a solution of approximately 0.1 mg **15** in 20 μL MeCN/acetic acid (1/1) and incubated at RT for 10 min. The product was diluted with 10 mL of water and loaded onto a C18 Sep Pak Plus cartridge, which had been preconditioned with 10 mL of MeOH and rinsed with 10 mL of water. The cartridge was then washed with 10 mL water, and the product was eluted in 300 to 500 μL fractions with a 1:1 mix (v/v) of EtOH/MeCN (2 mL). The radioactive fractions were evaporated to dryness, and the residue was dissolved in 200 μL TFA. After 30 min, the solvent was evaporated *in vacuo*. The crude product was isolated from unlabelled tributyltin precursor by RP-HPLC (20% to 40% B in 20 min) to afford the desired product (10.9 MBq). HPLC (20% to 40% B, 220 nm): *t*_*R*_ = 13.0 min, *K*′ = 6.22.

^*68*^*Ga****-****labelling*: A 1.25 mL fraction of ^68^Ge/^68^Ga generator (iTHEMBA Labs, South Africa) eluate (1 M HCl) was loaded onto a self-filled cartridge containing 300 mg SCX material (Bond Elut-SCX, Varian). The cartridge was then washed with water (1.0 mL) and purged with air. The ^68^Ga^3+^ was eluted with aq. NaCl (5 M, 0.5 mL) and HEPES (2.7 M aq., 90 or 140 μL) was added (pH 3.5 or 4.5, respectively). That solution was mixed with the precursor (3 nmol in 10 μL) and heated at 95°C for 5 min. After cooling, labelling efficiency and radiochemical purity were determined using Radio-TLC and Radio-HPLC. Radiochemical purity of all ^68^Ga-labelled conjugates was ≥95%. Therefore, the tracers were diluted and used *in vitro* experiments without further purification. Tracers for *in vivo* use were prepared in GMP-compliant procedure using a fully automated synthesis module (Scintomics GmbH, Germany) similar to the procedure described previously [33]. For animal studies, the EtOH for eluting the labelled tracer from a SPE cartridge was evaporated *in vacuo*.

^*177*^*Lu-labelling*: A 0.1 mM aqueous solution of chelator-coupled peptide (0.66 nmol) was added to 25 MBq [^177^Lu]LuCl_3_ (170 GBq/μmol, 17 GBq/mL 0.05 M HCl, IDB Radiopharmacy bv). The pH was adjusted to pH 5 by the addition of approximately 150 μL 0.1 M NH_4_OAc solution. After 45 min at 95°C, the labelling efficiency was examined by TLC and RP-HPLC.

### Determination of lipophilicity

To a solution of 0.5 to 1 MBq of radiolabelled peptide in 500 μl of phosphate-buffered saline (PBS, pH 7.4), 500 μl of n-octanol was added (*n* = 6). Vials were vortexed vigorously for 3 min. To achieve quantitative phase separation, the vials were centrifuged at 6,000 g for 5 min in a Biofuge 15 (Heraeus Sepatech, Osterode, Germany). The activity concentrations in 100 μl-samples of both the aqueous and the organic phase were measured in a γ-counter. Both the partition coefficient *P*_(o/w)_, which is defined as the molar concentration ratio of a single species A between n-octanol and water at equilibrium, and log*P*_(o/w)_, which is an important parameter used to characterize lipophilicity of a compound, were calculated.

### Metabolite analysis

^177^Lu-**10** and ^177^Lu-**11** were incubated in 80 μL of human serum (37°C) and 80 μL 0.4 M DTPA solution (RT), respectively and after 1, 24 and 48 h, the stability was determined by TLC analysis. The ^68^Ga-labelled tracers (40 to 45 MBq) were injected into the tail vein of CD-1 nu/nu mice. The animals were sacrificed after 30 min, and urine, blood and kidney samples were taken. The kidney was frozen in liquid nitrogen, homogenized with a ball mill and extracted with 0.2 to 1 mL PBS containing 200 nmol 2-(phosphonomethyl)pentane-1,5-dioic acid (PMPA). The suspension was first centrifuged (15,000 g), and after ultrafiltration, the extracts were analysed by RP-HPLC. The blood samples were centrifuged to separate the plasma from the blood cells. Plasma proteins were removed by precipitation with acetonitrile (10 min, 4°C) and subsequent centrifugation and ultrafiltration. The blood extract was also analysed by RP-HPLC.

### *In vitro* assays

*Cell culture*: PSMA^+^ LNCaP cells (CLS: 300265) were grown in DMEM/Nutrition Mix F-12 with Glutamax-I (1:1) (Invitrogen, Life Technologies, Darmstadt, Germany) supplemented with 10% FCS. Cultures were maintained at 37°C in a 5% CO_2_/humidified air atmosphere. One day prior to the experiment, cells were harvested using Trypsin/EDTA (0.05% and 0.02%) in PBS, centrifuged and resuspended with culture medium. For cell counting, a Countesse automated cell counter (Invitrogen, Carlsbad, USA) was used. All *in vitro* binding and internalization studies were performed using live cells seeded 1 day prior to the experiment. For IC_50_ determination, 150,000 cells/mL were transferred to 24-well plates (1 mL/well), and for internalization studies, 125,000 cells/mL were transferred into PLL-coated 24-well plates.

*Determination of IC*_*50*_: The culture medium was removed, and the cells were washed once with 500 μL of HBSS (Hank's balanced salt solution, Biochrom, Berlin, Germany, containing 1% bovine serum albumin (BSA)), before being left to equilibrate in 200 μL of HBSS (1% BSA) on ice for 15 min. Then, 25 μL/well of either HBSS (1% BSA; control) or of solutions containing the respective unlabelled ligand of interest in increasing concentrations (10^-10^-10^-4^ M in HBSS (1% BSA)) was added, followed by the addition of 25 μL of ([^125^I]I-BA)KuE in HBSS (1% BSA). Experiments were carried out in triplicate for each concentration. The final radioligand concentration was 0.2 nM in all binding assays. Cells were incubated on ice for 60 min. Incubation was terminated by the removal of the incubation medium. Cells were thoroughly rinsed with 250 μL of HBSS. The wash medium was combined with the supernatant of the previous step. This fraction represents the amount of free radioligand. Cells were then lysed using 250 μL of 1 N NaOH, the lysate was transferred to vials and combined with 250 μL of HBSS used for rinsing the wells. Quantification of the amount of free and bound activity was performed in a γ-counter.

*Internalization studies*: The culture medium was removed, and the cells were washed once with 500 μL DMEM-F12 (5% BSA) before being left to equilibrate in 200 μL DMEM-F12 (5% BSA) at 37°C for a minimum of 15 min. Then, 25 μL (per well) of either DMEM-F12 (5% BSA) or of a 100-μM PMPA solution (blocking) was added, followed by the addition of 25 μL of ^68^Ga/^177^Lu-labelled PSMA ligand. The final radioligand concentration was 0.2/0.5 nM in all internalization assays. To determine internalization kinetics, cells were then incubated at 37°C for 5, 15, 30 and 60 min, respectively. Experiments were carried out in triplicate for each time point (control and blocking). Incubation was terminated by placing the plate on ice for approximately 1 min and by subsequent removal of the incubation medium. Cells were thoroughly rinsed with 250 μL of PBS. The wash medium was combined with the supernatant of the previous step. This fraction represents the amount of free radioligand. To remove receptor surface bound radioactivity, the cells were then incubated for 10 min with 250 μL of ice cold PMPA solution (10 μM in PBS). After removal of the supernatant, the cells were thoroughly rinsed with another 250 μL of ice cold PBS. Both fractions were combined. The internalized activity was released by incubation with 250 μL of 1 N NaOH, transferred to vials and combined with 250 μL of 1 N NaOH used for rinsing the wells. Quantification of the amount of free, PMPA-releasable and internalized activity was performed in a γ-counter.

### *In vivo* experiments

All animal experiments were conducted in accordance with general animal welfare regulations in Germany (Deutsches Tierschutzgesetz, approval #55.2-1-54-2532-71-13). The prostate cancer cell line LNCaP was suspended 1/1 in serum-free medium and Matrigel (BD Biosciences, Germany) and approximately 10^7^ cells in 200 μL were inoculated subcutaneously on the right shoulder of 6 to 8 weeks old CD-1 nu/nu mice (Charles River Laboratories). Tumours were grown for 2 to 4 weeks (males) and 4 to 6 weeks (females) to reach 4 to 8 mm in diameter.

*Biodistribution*: The ^68^Ga-labelled PSMA ligands (approximately 7 to 15 MBq, 0.1 to 0.2 nmol) were injected into the tail vein of isoflurane anesthetized animals. The organ distribution was examined 1 h p.i. and quantified in a γ-counter.

*μPET imaging*: Imaging studies were performed at a Siemens Inveon small animal PET, followed by data analysis using the Inveon Research Workplace software. The animals were anesthesized with isoflurane and injected via tail vein with 14 to 18 MBq (0.2 nmol) of tracer. Dynamic imaging was performed after on-bed injection for 1.5 h. Static images were recorded at 1 h p.i. with an acquisition time of 15 min. For the blockade image, animals were coinjected with 8 mg/kg of PMPA. Images were reconstructed using 3D ordered-subsets expectation maximum (OSEM3D) algorithm without scanner and attenuation correction.

## Results

### Chemistry

**Figure 3:**
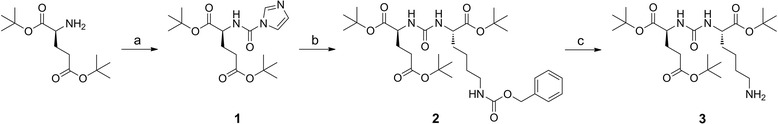
**Synthesis of the PSMA binding motif 3. (a)** DCI, TEA, DMAP [DCM]; **(b)** H-Lys(Z)-OtBu, TEA [DCE]; **(c)** Pd/C (10%), H_2_ [EtOH].

The *t*Bu-protected PSMA binding motif ((OtBu)KuE(OtBu)_2_, **3**) was synthesized in 73% yield via three steps of solution phase chemistry as previously described with minor modifications (Figure 3, [12]).

**Figure 4:**
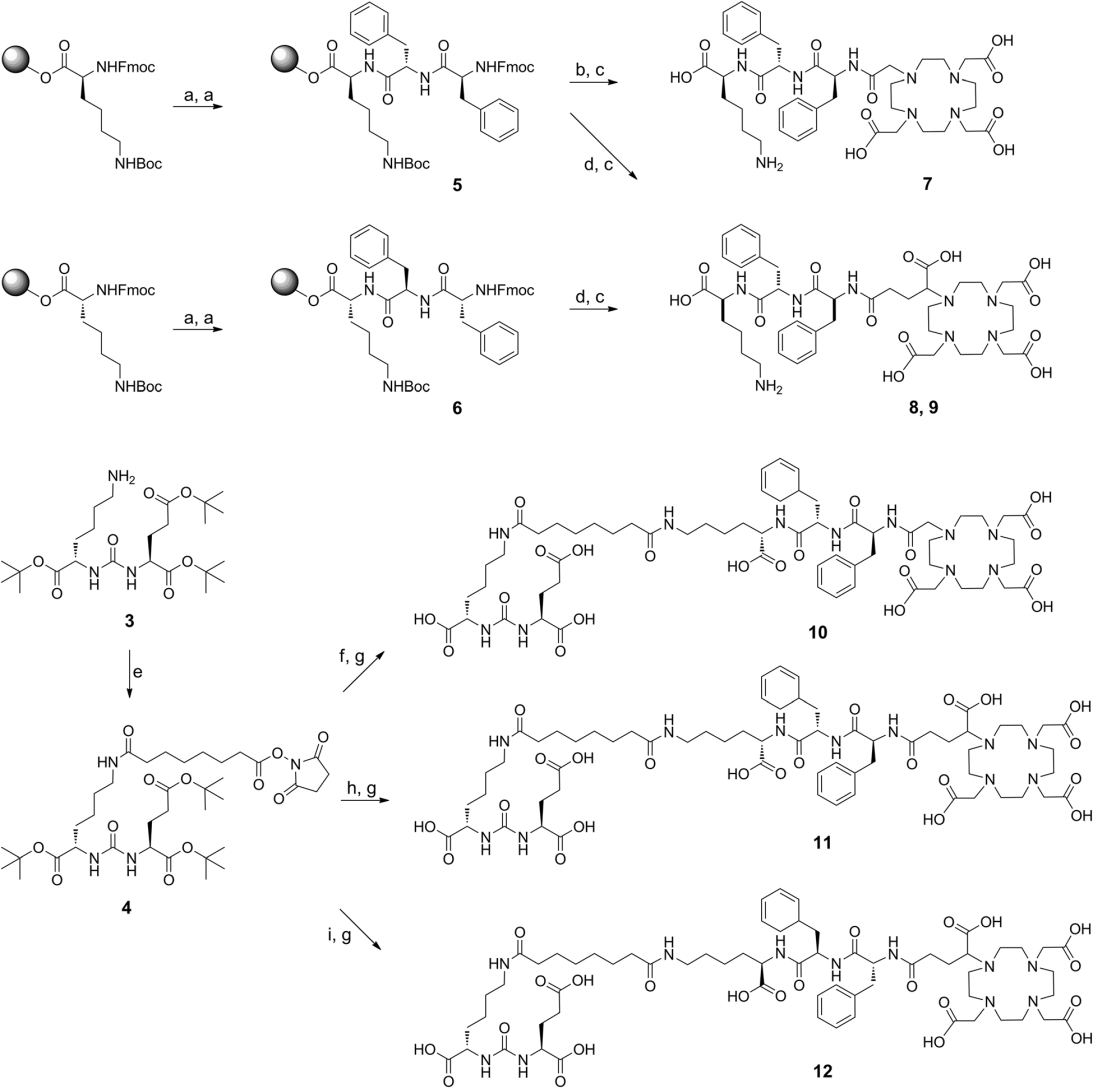
**Modified solid**-**phase synthesis of the DOTA conjugate 10** **[** 17 **]** **and DOTAGA conjugates 11 and 12. (a)** 20% piperidine in NMP, Fmoc-Phe-OH, HOBt, TBTU, DIPEA, [NMP]; **(b)** 20% piperidine in NMP, DOTA-tris-*t*Bu-ester, HATU, DIPEA [NMP]; **(c)** 95% TFA, 2.5% TIBS, 2.5% H_2_O; **(d)** 20% piperidine in NMP, DOTAGA-anhydride, DIPEA [NMP]; **(e)** DSS, TEA [DMF]; **(f) 7**, TEA [DMF]; **(g)** TFA; **(h) 8**, TEA [DMF]; **(i) 9**, TEA [DMF].

The ligands DOTA-FFK(Sub-KuE) (**10**), DOTAGA-FFK(Sub-KuE) (**11**) and DOTAGA-ffk(Sub-KuE) (**12**) were prepared according to Figure 4. The peptidic spacers H-l-Phe-l-Phe-l-Lys(Boc)-OH (FFK(Boc), **5**) and H-d-Phe-d-Phe-d-Lys(Boc)-OH (ffk(Boc), **6**) were synthesized via solid phase peptide synthesis using a standard Fmoc protocol. The prochelators DOTA-tris-*t*Bu-ester and DOTAGA-anhydride were coupled to the resin-bound peptides in almost quantitative yields. Subsequently, the chelator-conjugated peptides were cleaved from the resin (TFA/TIBS/H_2_O) and simultaneously deprotected to yield **7**, **8** and **9**. The *t*Bu-protected PSMA binding motif **3** was first reacted with DSS in solution and then coupled with the chelator-functionalized peptides **7**, **8** and **9**. After acidic cleavage of the remaining *t*Bu-protecting groups, the final products were purified by RP-HPLC. The products were obtained in approximately 15% yield (based on tripeptide). The identity of **10**, **11** and **12** was confirmed by MS.

The ^nat^Ga^III^ and ^nat^Lu^III^ complexes of compounds **10**, **11** and **12** were prepared by incubation of **10**, **11** and **12** with an equimolar amount of aq. Ga(NO_3_)_3_ at 40°C for 30 min and an 2.5-molar excess of LuCl_3_ at 95°C for 30 min, respectively. Quantitative metal complex formation was confirmed by RP-HPLC and MS.

### Radiochemistry

([^125^I]I-BA)KuE, the reference radioligand in all PSMA binding assays carried out in this study, was synthesized in solution starting from 4-iodo-benzoic acid according to methods described in the literature [12,31,32]. The respective Bu_3_Sn-precursor **13** was destannylated with [^125^I]NaI using peracetic acid as the oxidizing agent within 10 min at RT. After cartridge purification subsequent *t*Bu-deprotection and RP-HPLC purification, the final product was obtained in a radiochemical yield of 44 ± 5% and radiochemical purity of >99%. The reference material for chromatographic analyses (cold) (I-BA)KuE was synthesized by coupling 4-iodo-benzoic acid to **3**, subsequent *t*Bu-deprotection and RP-HPLC purification.

Preparation of ^68^Ga for labelling of **10**, **11** and **12** was performed by combination of previously described methods [34,35] with minor modifications. The ^68^Ga^3+^ eluted with 1 M HCl from a ^68^Ge/^68^Ga generator (iThemba Labs, South Africa) was retained on a strong cation exchange cartridge followed by elution with 0.5 mL 5 M NaCl, resulting in highly concentrated ^68^Ga activity. Quantitative ^68^Ga-complexation was achieved using 3 nmol of the respective DOTA- or DOTAGA-conjugated PSMA inhibitors (95°C, 5 min, pH adjusted to 3.5 and 4.5 by addition of 2.7 M HEPES, respectively), allowing their use in *in vitro* studies without further purification. Specific activities of the ^68^Ga-labelled PSMA inhibitors were 250 to 300 GBq/μmol. The ^68^Ga-labelling for *in vivo* biodistribution and PET imaging studies were carried out using a fully automated GMP-compliant procedure using a GRP synthesizer (Scintomics GmbH, Germany) [33]. In these cases, the obtained specific activities were 80 to 120 GBq/μmol.

To obtain RCY >95% for complexation of **10**, **11** and **12** with [^177^Lu]LuCl_3_, 0.66 nmol of precursor were reacted with 25 MBq [^177^Lu]LuCl_3_ (*A*_S_ = 170 GBq/μmol) at pH 5 (0.1 M NH_4_OAc, 95°C, 45 min) resulting in specific activities of *A*_S_ ≥38 GBq/μmol.

### PSMA binding affinity

The PSMA binding affinities were determined in a competitive binding assay using LNCaP human prostate carcinoma cells and the known high affinity PSMA ligand ([^125^I]I-BA)KuE [10] (*c* = 0.2 nM) as the radioligand. The IC_50_ values for the metal-free PSMA-inhibiting compounds and their respective ^nat^Ga and ^nat^Lu complexes are summarized in Table 1. HBED-CC-Ahx-KuE and [^nat^Ga]HBED-CC-Ahx-KuE [12] were included in this study as reference compounds. The PSMA affinities of **11** and **12**, as well as their respective ^nat^Ga and ^nat^Lu analogues were consistently higher than the affinities of the respective DOTA constructs (**10**). Substitution of the l-amino acids in the linker region of the ligands by d-amino acids showed only a negligible effect on PSMA affinity (**12** vs **11**).

**Table 1:** **PSMA affinities** (**IC**_**50**_) **of the compounds investigated**

**Ligand**		**IC**_**50**_ **[nM]**
HBED-CC-Ahx-KuE		5.7 ± 0.5
[^nat^Ga]HBED-CC-Ahx-KuE		6.1 ± 0.8
DOTA-FFK(Sub-KuE)	(**10**)	13.1 ± 2.3
[^nat^Ga]DOTA-FFK(Sub-KuE)	(^nat^Ga-**10**)	29.5 ± 6.6
[^nat^Lu]DOTA-FFK(Sub-KuE)	(^nat^Lu-**10**)	54.7 ± 6.1
DOTAGA-FFK(Sub-KuE)	(**11**)	10.2 ± 1.5
[^nat^Ga]DOTAGA-FFK(Sub-KuE)	(^nat^Ga-**11**)	12.1 ± 3.9
[^nat^Lu]DOTAGA-FFK(Sub-KuE)	(^nat^Lu-**11**)	15.1 ± 1.5
DOTAGA-ffk(Sub-KuE)	(**12**)	13.9 ± 0.4
[^nat^Ga]DOTAGA-ffk(Sub-KuE)	(^nat^Ga-**12**)	15.9 ± 0.5
[^nat^Lu]DOTAGA-ffk(Sub-KuE)	(^nat^Lu-**12**)	13.1 ± 2.2

Binding assays were performed using LNCaP cells (150,000/well) and ([^125^I]I-BA)KuE (c = 0.2 nM) as the radioligand. Cells were incubated in HBSS (1% BSA) at 4°C for 1 h. Data are expressed as mean ± SD (*n* = 3).

### Internalization kinetics and specificity of cell binding

The cellular uptake and internalization kinetics of the different ^68^Ga- and ^177^Lu-labelled PSMA ligands was determined using PSMA-expressing LNCaP cells. In the case of the ^68^Ga-labelled compounds, the final peptide concentration in the assays was 0.2 nM. Since all ^177^Lu analogues were obtained in significantly lower specific activities than the ^68^Ga analogues, the ligand concentration had to be increased to 0.5 nM to obtain reasonable count rates for assay evaluation. All internalization studies were accompanied by reference studies using ([^125^I]I-BA)KuE (*c* = 0.2 nM). This experimental setup allows data normalization and eliminates the influence of cell count and cell viability on absolute cellular tracer uptake. To investigate the specificity of binding to PSMA, experiments were also carried out in the presence of 10 μM PMPA, a known high-affinity PSMA inhibitor (1.4 nM, [7]). Furthermore, to be able to discriminate between total cellular activity (sum of membrane associated and internalized activity) and internalized activity, all incubations were followed by a washing step with 10 μM PMPA at 4°C to remove specifically cell-surface bound radioligand by displacement.

**Figure 5:**
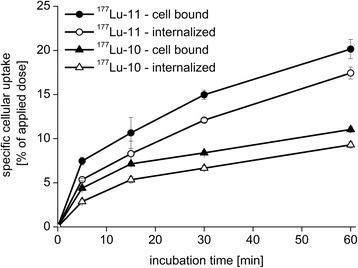
**Cell binding and internalization kinetics of** ^**177**^ **Lu-** **10 and** ^**177**^ **Lu**-**11 in LNCaP cells.** In PLL-coated plates, 125,000 cells/well were incubated with the respective radioligand (*c* = 0.5 nM) at 37°C in DMEM-F12 (5% BSA). The total cellular activity was corrected for non-specific binding (10 μM PMPA). All data are expressed as mean ± SD (*n* = 3).

Non-specific binding in the presence of 10 μM PMPA was lower than 0.5% for all compounds investigated. As expected, the cellular binding and internalization of all radiometalated PSMA ligands in this study increased over the time of observation (1 h). For all compounds, the fraction of internalized activity ranged between 4.5 ± 0.2% and 27.7 ± 1.6% of the total added activity after 1 h. The fraction of non-internalized, i.e. membrane-bound activity was 1% to 3% in all experiments (exemplarily shown for ^177^Lu-**10** and ^177^Lu-**11** in Figure 5).

Variations in cell count and/or cell viability between experiments can significantly affect the cellular tracer uptake but cannot be fully avoided. Therefore, internalization data (Table 2) are normalized to the uptake of ([^125^I]I-BA)KuE, that was always assayed in parallel as an external reference. All DOTA and DOTAGA-coupled ligands showed a lower internalization efficiency compared to the radioiodinated reference compound, whereas [^68^Ga]HBED-CC-Ahx-KuE was internalized almost to the same degree as ([^125^I]I-BA)KuE.

**Table 2:** **Internalization of radiometalated ligands in percentage of external reference** ([^**125**^ **I**]**I**-**BA**)**KuE**

**Ligand**		**Internalization** **(** **1 h** **) ** **in** **% ** **of** **([** ^**125**^ **I** **]** **I** **-** **BA** **)** **KuE**
[^68^Ga]HBED-CC-Ahx-KuE		91.1 ± 1.7
[^68^Ga]DOTA-FFK(Sub-KuE)	(^68^Ga-**10**)	14.6 ± 0.8
[^177^Lu]DOTA-FFK(Sub-KuE)	(^177^Lu-**10**)	19.3 ± 0.9
[^68^Ga]DOTAGA-FFK(Sub-KuE)	(^68^Ga-**11**)	28.4 ± 0.7
[^177^Lu]DOTAGA-FFK(Sub-KuE)	(^177^Lu-**11**)	36.1 ± 1.1
[^68^Ga]DOTAGA-ffk(Sub-KuE)	(^68^Ga-**12**)	42.5 ± 1.7
[^177^Lu]DOTAGA-ffk(Sub-KuE)	(^177^Lu-**12**)	44.4 ± 1.8

Data is corrected for unspecific binding, *c* = 0.2 nM for ^68^Ga, *c* = 0.5 nM for ^177^Lu compounds, 37°C, 1 h, 125,000 cells/well, PLL-coated plates.

### Determination of lipophilicity

The partition coefficient log*P* between n-octanol and PBS was determined for the compounds listed in Table 3 using the shake flask method. All compounds are highly hydrophilic, with the DOTAGA compounds being up to one order of magnitude more hydrophilic than the corresponding DOTA analogues. Amongst all compounds tested, [^68^Ga]HBED-CC-Ahx-KuE was the most hydrophilic compound.

**Table 3:** **Lipophilicity of the radiolabelled ligands** (**log** ***P***_(**o**/**w**)_; **distribution coefficient in n**-**octanol**/**PBS**)

**Ligand**		**log** ***P***_**(o/w)**_
[^68^Ga]HBED-CC-Ahx-KuE		−4.1 ± 0.1
[^68^Ga]DOTA-FFK(Sub-KuE)	(^68^Ga-**10**)	−3.1 ± 0.2
[^177^Lu]DOTA-FFK(Sub-KuE)	(^177^Lu-**10**)	−2.7 ± 0.02
[^68^Ga]DOTAGA-FFK/ffk(Sub-KuE)	(^68^Ga-**11**/**12**)^a^	−3.6 ± 0.1
[^177^Lu]DOTAGA-FFK/ffk(Sub-KuE)	(^177^Lu-**11**/**12**)^a^	−3.9 ± 0.1

Data are expressed as mean ± SD (*n* =6). ^a^Determined for ^68^Ga-**11** and ^177^Lu-**11**.

### Metabolite analysis

The stability of ^177^Lu-**10** and ^177^Lu-**11** was investigated by incubation in human serum for 48 h at 37°C and by DTPA challenge (0.4 M, RT, 48 h) and subsequent TLC analysis. Release of ^177^Lu^III^ from the complexes was not observed under the respective experimental conditions in the examined time frame.

**Figure 6:**
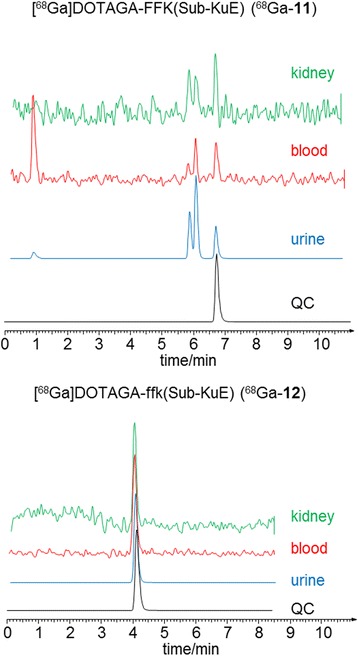
**Exemplary radio**-**HPLC analyses of extracts from homogenized organs and body fluids.** HPLC-traces of CD-1 nu/nu mice (0.5 h p.i. of 40 to 45 MBq of ^68^Ga labelled tracer, Chromolith column, flow rate 3 mL/min) for ^68^Ga-**11** (3% for 3 min, 3% to 95% in 6 min, 95% for 3 min) and ^68^Ga-**12** (3% to 95% in 6 min, 95% for 3 min).

To investigate the influence of the amino acid composition of the spacer (KFF vs kff) on the metabolic stability *in vivo*, tissue homogenates and body fluids of mice injected with ^68^Ga-**11** and ^68^Ga-**12** were analysed by radio-HPLC. Representative HPLC profiles of extracts and body fluids are shown in Figure 6. Extraction efficiencies from the blood and from the kidney were 56% and 43% for ^68^Ga-**11** and 61% and 62% for ^68^Ga-**12**, respectively. Rapid *in vivo* degradation was observed for ^68^Ga-**11** consisting of the l-amino acid tripeptide FFK, resulting in only 21% intact tracer in blood at 0.5 h. ^68^Ga-**12** that consists of the d-amino acid tripeptide ffk, was found to be stable in blood (100% intact tracer) at 0.5 h p.i..

### Biodistribution and small-animal PET studies

**Figure 7:**
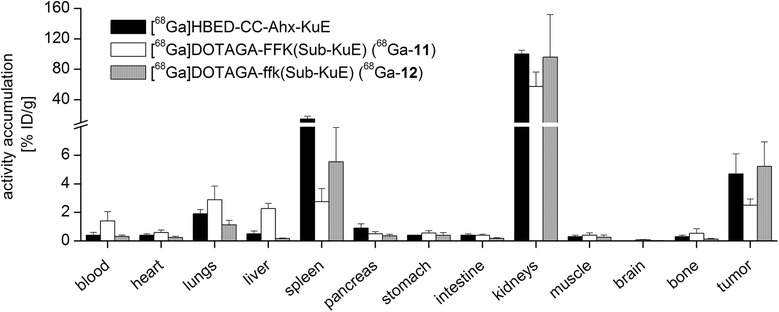
**Biodistribution data** **(in %** **ID/** **g)** **at 1 h p.i..** The biodistribution of ^68^Ga-**12** in LNCaP tumour xenograft bearing CD-1 nu/nu mice (*n* =5) in comparison to ^68^Ga-**11** (*n* = 5) and [^68^Ga]HBED-CC-Ahx-KuE (*n* = 4).

A comparison of the biodistribution of compounds ^68^Ga-**11**, ^68^Ga-**12** and the reference [^68^Ga]HBED-CC-Ahx-KuE in LNCaP-tumour-bearing CD-1 nu/nu mice (1 h p.i.) is shown in Figure 7.

**Figure 8:**
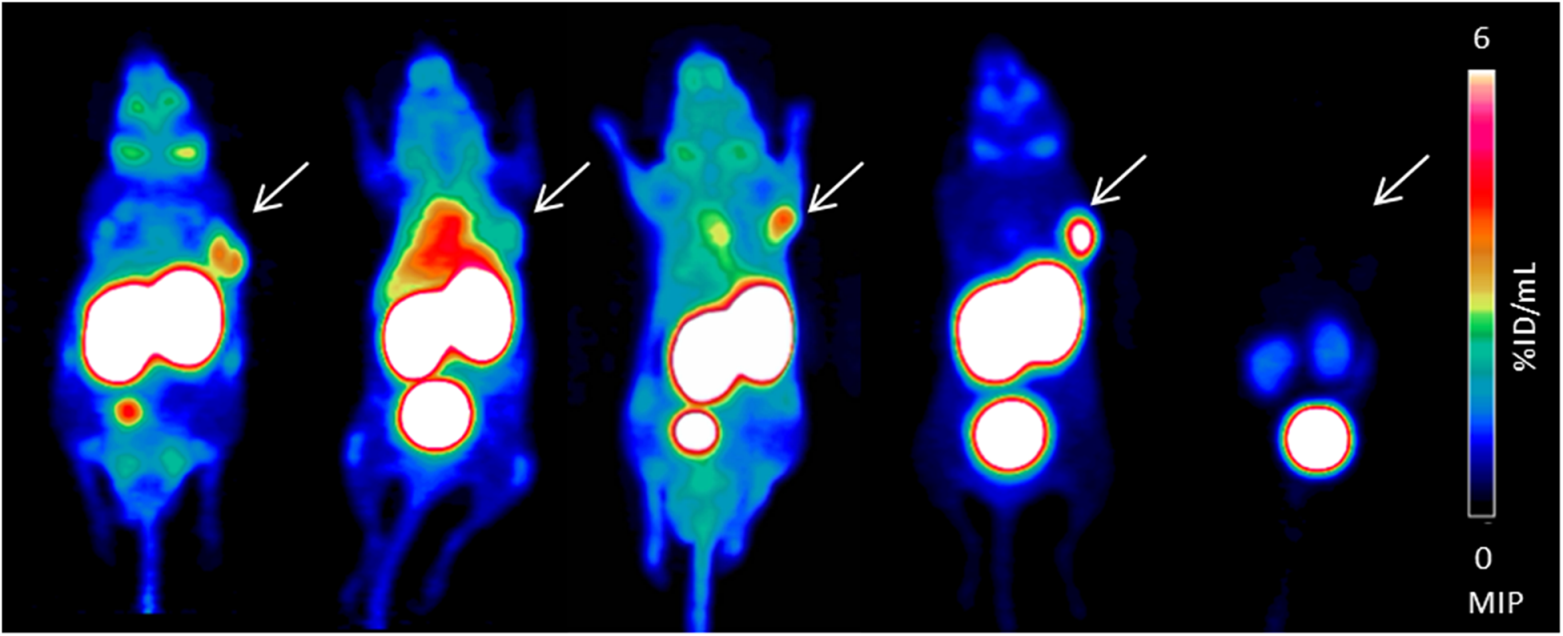
**Maximum intensity projections** **(MIP)** **of μPET scans.** MIP (1 h p.i. for 15 min, 0% to 6% ID/mL) of five LNCaP human prostate carcinoma xenograft bearing mice (right shoulder) after injection of approximately 15 MBq **(a)** [^68^Ga]HBED-CC-Ahx-KuE, **(b)** ^68^Ga-**10**, **(c)** ^68^Ga-**11**, **(d)** ^68^Ga-**12**, **(d′)** ^68^Ga-**12** + blocking (8 mg/kg PMPA).

Comparative static PET scans were performed with ^68^Ga-**10**, ^68^Ga-**11**, ^68^Ga-**12** and [^68^Ga]HBED-CC-Ahx-KuE in the same animal model for 15 min at 1 h p.i.. The maximum intensity projections depicted in Figure 8 confirm higher tumour-to-background ratios for ^68^Ga-**11** (c) compared to the respective DOTA analogue ^68^Ga-**10** (b). In agreement with the results obtained from the biodistribution (Figure 7), the metabolically stable ^68^Ga-**12** (d) exhibits increased tumour accumulation and a lower unspecific whole body uptake and tumour-to-background ratios even higher than those of the reference compound [^68^Ga]HBED-CC-Ahx-KuE (a). That tracer uptake into tumour and kidneys is specific and PSMA mediated [18], as illustrated by the blocking experiment with PMPA (8 mg/kg) (Figure 8d′).

**Figure 9:**
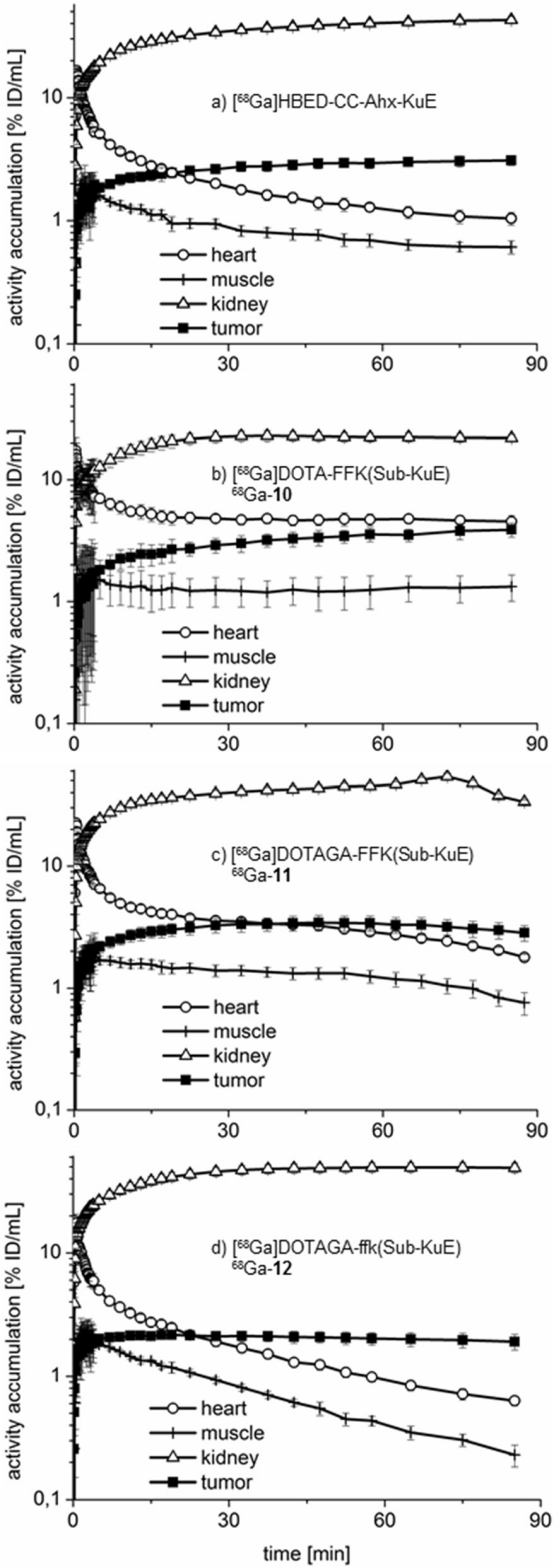
**Time** **-activity-** **curves** **(logarithmic plot)** **in** **% ID** **/mL for blood pool** **(heart),** **muscle,** **kidney and tumour.** Graphs for **(a)** [^68^Ga]HBED-CC-Ahx-KuE, **(b)** ^68^Ga-**10**, **(c)** ^68^Ga-**11**, **(d)** ^68^Ga-**12** are derived from dynamic small animal PET data.

In addition, the biodistribution kinetics of ^68^Ga-**10**, ^68^Ga-**11**, ^68^Ga-**12**, and the reference compound [^68^Ga]HBED-CC-Ahx-KuE were investigated by carrying out dynamic μPET scans over a period of 1.5 h in CD-1 nu/nu mice bearing LNCaP tumour xenografts (Figure 9). Despite the somewhat lower individual tumour uptake of ^68^Ga-**12** observed in this μPET-study, the markedly enhanced clearance of this compound from non-target tissues resulted in higher tumour-to-tissue ratios for ^68^Ga-**12** compared to [^68^Ga]HBED-CC-Ahx-KuE.

## Discussion

Due to the consistent overexpression of PSMA in especially hormone-refractory and metastatic prostate cancer, this cell surface enzyme represents an excellent target for high-contrast PET imaging and also for therapeutic applications. The currently available PSMA-targeted radiopharmaceuticals are highly optimized either for imaging [36,37] or endoradiotherapeutic applications [25], but so far none of the compounds was evaluated for a theranostic concept. Our approach was therefore focused on the development of a PSMA ligand allowing for both, complexation with, e.g. ^68^Ga or ^111^In for PET or SPECT and with therapeutic M^3+^-radiometals, e.g. ^177^Lu, ^90^Y or ^225^Ac for endoradiotherapy. The most frequently used chelator for this purpose is DOTA, and recently, a first KuE-based DOTA-conjugated PSMA inhibitor (^68^Ga-**10**, [17]) has been described. In a comparative preclinical evaluation with [^68^Ga]HBED-CC-Ahx-KuE [18], ^68^Ga-**10** showed significantly lower tumour uptake than [^68^Ga]HBED-CC-Ahx-KuE. However, since ^68^Ga-**10** may be labelled with a broad palette of diagnostic and therapeutic radiometals, whereas [^68^Ga]HBED-CC-Ahx-KuE may not, we used ^68^Ga-**10** as a starting point for the development of improved PSMA-targeted theranostics.

In a first attempt to improve the pharmacokinetics of the DOTA-coupled PSMA ligand **10**, we increased the hydrophilicity of the ligand by substitution of DOTA by DOTAGA, resulting in **11**. Although [^68^Ga]HBED-CC-Ahx-KuE contains the lipophilic HBED-CC chelator, which improves binding affinity [18], a log*P* of -4.1 ± 0.1 was determined, revealing a significantly higher hydrophilicity compared to log*P* = -3.1 ± 0.2 for ^68^Ga-**10**. The hydrophilicity of the ^68^Ga-labelled DOTAGA-coupled ligand **11** increased to log *P* = -3.6 ± 0.1. For ^68^Ga, as well as ^177^Lu labelling, the DOTA-conjugated ligand **10** and the DOTAGA-conjugated ligands **11** and **12** exhibited no differences in labelling efficiency.

To examine the metabolic stability of these new PSMA tracers *in vivo*, CD-1 nu/nu mice were injected with the ^68^Ga-labelled ligand **11**. Due to its l-amino acid spacer, ^68^Ga-**11** was rapidly metabolized. Furthermore, we demonstrated that fast metabolization of ^68^Ga-**10** and ^68^Ga-**11** resulted in discontinuous clearance kinetics and low tumour accumulation *in vivo* (Figure 7, 8, 9). This most likely explains the finding in the literature, that despite moderate affinity towards PSMA (IC_50_ = 29.5 ± 6.6 nM) the DOTA ligand ^68^Ga-**10** exhibited unfavourable tumour targeting compared to [^68^Ga]HBED-CC-Ahx-KuE. Further, we observed significant inter-individual differences in metabolization kinetics for ^68^Ga-**11** (with its l-amino acid spacer) in mice, which might explain inconsistent *in vivo* results obtained with PSMA ligands with l-amino acid spacers [38,39]. To overcome the problem of rapid proteolytic cleavage of radiolabelled **11**, we substituted the l-amino acid spacer (FFK) by its d-amino acids counterpart ffk (**12**) resulting in high metabolic stability *in vivo*.

In addition to the improvement in metabolic stability for **12**, we found that the DOTAGA-conjugated ligands **11** and **12**, as well as their gallium(III) and lutetium(III) complexes, showed a significantly increased affinity towards PSMA on LNCaP cells compared to the DOTA-coupled ligand **10**/^nat^Ga-**10**/^nat^Lu-**10**. Especially, ^nat^Lu-**10** (IC_50_ = 54.7 ± 6.1 nM) was less affine compared to ^nat^Lu-**11** (15.1 ± 1.5 nM) or ^nat^Lu-**12** (13.1 ± 2.2 nM). This finding most likely is due to the increased charge of the DOTAGA-ligands. In our competitive binding assay, unlike reported for other assays in the literature [18], a significantly lower affinity for ^nat^Ga-**10** (IC_50_ of 29.5 ± 6.6 nM) was determined compared to [^nat^Ga]HBED-CC-Ahx-KuE (IC_50_ = 6.1 ± 0.8 nM).

As expected from their enhanced PSMA affinities, the DOTAGA ligands ^68^Ga/^177^Lu-**11** and ^68^Ga/^177^Lu-**12** showed up to twofold increased internalization compared to the corresponding DOTA ligands ^68^Ga/^177^Lu-**10**. Interestingly, ^68^Ga/^177^Lu-**12** with the ffk spacer showed a higher internalization rate than ligand **11** with the FFK spacer. Blocking experiments with PMPA highlight the high PSMA-specificity of these tracers.

Accordingly, the pharmacokinetics and tumour uptake of the new ligands, i.e. ^68^Ga-**12** in LNCaP-tumour bearing CD-1 nu/nu mice could be enhanced (Figure 7). The metabolically instable ^68^Ga-**11** revealed higher activity levels in the blood pool and almost all organs, whereas the metabolically stable tracer ^68^Ga-**12** displayed no significant uptake in the gastrointestinal tract (<0.5% ID/g) and high kidney accumulation of 96 ± 45% ID/g. The kidney uptake is PSMA-specific, as shown in the blocking experiment (Figure 8d′). Although [^68^Ga]HBED-CC-Ahx-KuE showed a higher affinity and increased internalization rate compared to ^68^Ga-**12**, the *in vivo* biodistribution (Figure 7) and μPET imaging studies (Figure 8) revealed favourable *in vivo* characteristics for ^68^Ga-**12**. The uptake of ^68^Ga-**12** into the PSMA positive LNCaP tumour xenograft was somewhat increased compared to that of [^68^Ga]HBED-CC-Ahx-KuE (5.2 ± 1.2% vs. 4.7 ± 0.2% ID/g, respectively). Further, [^68^Ga]HBED-CC-Ahx-KuE showed enhanced tracer uptake into the lung (1.9 ± 0.4% vs. 1.1 ± 0.3% ID/g), liver (0.5 ± 0.1 vs. 0.2 ± 0.04% ID/g), spleen (14.7 ± 2.6 vs. 5.5 ± 0.2% ID/g) and pancreas (0.9 ± 0.2 vs. 0.4 ± 0.1% ID/g). Therefore, the lower unspecific uptake of ^68^Ga-**12** compared to [^68^Ga]HBED-CC-Ahx-KuE resulted in higher tumour-to-background ratios, i.e. tumour-to-blood (17.0 ± 0.5 vs. 12.5 ± 0.7) and tumour-to-skeletal muscle (19.8 ± 0.7 vs. 16.2 ± 0.1).

After the initial distribution phase of a radiopharmaceutical, straightforward clearance kinetics is typically characterized by a linear decrease in a semilogarithmic plot (two-compartment model). Interestingly, this kinetic profile (Figure 9) was only observed for ^68^Ga-**12**, whereas all other tracers (or their radiolabelled metabolites) exhibit unspecific retention or redistribution effects, resulting in non-linear kinetics (e.g. between 10 and 60 min) in the semilogarithmic plot. Considering the time-activity curves, it is worth noting that based on the kinetics of the reference compound [^68^Ga]HBED-CC-Ahx-KuE redistribution from tissues and compartments with unspecific uptake in the early phase seems likely and might explain the discontinuous clearance and increasing tumour uptake at later time points. The uptake of DOTA conjugate ^68^Ga-**10** in the tumour is continuously increasing over time, but surprisingly without a corresponding decline of activity in muscle or blood (as measured over the heart). In contrast, a slow washout from the target and non-target tissues was observed for ^68^Ga-**11**. Most likely as a result of its metabolic instability, ^68^Ga-**11** exhibited discontinuous and only moderate clearance kinetics. However, ^68^Ga-**12** shows typical and nearly ideal *in vivo* pharmacokinetics, both for the target tissue (tumour and kidney) as well as the non-target tissue muscle and the blood. Equivalent to our DOTAGA ligand **12**, the biodistribution of a naphthyl-containing [^177^Lu]DOTA-PSMA ligand was described recently [40], therefore, we assume that ^177^Lu-**12** also reveals favourable properties *in vivo*.

## Conclusions

Compared to the metabolically unstable DOTA conjugate **10** and DOTAGA-conjugate **11**, ^68^Ga- and ^177^Lu-**12** were found to be rapidly internalized into PSMA expressing cells, to have a favourable pharmacokinetic profile *in vivo* and only negligible unspecific uptake with no redistribution *in vivo*. Based on the DOTAGA chelator and the use of a d-amino acid spacer, compound **12** possesses remarkable potential for a PSMA based theranostics concept and is a suitable lead structure for the continuing development of PSMA ligands with further improved affinity.
